# Targeting KRAS mutant cancers: from druggable therapy to drug resistance

**DOI:** 10.1186/s12943-022-01629-2

**Published:** 2022-08-04

**Authors:** Chunxiao Zhu, Xiaoqing Guan, Xinuo Zhang, Xin Luan, Zhengbo Song, Xiangdong Cheng, Weidong Zhang, Jiang-Jiang Qin

**Affiliations:** 1 The Cancer Hospital of the University of Chinese Academy of Sciences (Zhejiang Cancer Hospital), Institute of Basic Medicine and Cancer (IBMC), Chinese Academy of Sciences, Hangzhou, 310022 China; 2 School of Molecular Medicine, Hangzhou Institute for Advanced Study, UCAS, Hangzhou, 310024 China; 3 Key Laboratory of Prevention, Diagnosis, and Therapy of Upper Gastrointestinal Cancer of Zhejiang Province, Hangzhou, 310022 China; 4 College of Pharmaceutical Science, Zhejiang University of Technology, Hangzhou, 310032 China; 5 Institute of Interdisciplinary Integrative Medicine Research and Shuguang Hospital, Shanghai University of Traditional Chinese Medicine, Shanghai, 201203 China; 6 School of Pharmacy, Second Military Medical University, Shanghai, 200433 China

**Keywords:** KRAS mutations, Druggable, Resistance, Combination therapy

## Abstract

Kirsten Rat Sarcoma Viral Oncogene Homolog (KRAS) is the most frequently mutated oncogene, occurring in a variety of tumor types. Targeting KRAS mutations with drugs is challenging because KRAS is considered undruggable due to the lack of classic drug binding sites. Over the past 40 years, great efforts have been made to explore routes for indirect targeting of KRAS mutant cancers, including KRAS expression, processing, upstream regulators, or downstream effectors. With the advent of KRAS (G12C) inhibitors, KRAS mutations are now druggable. Despite such inhibitors showing remarkable clinical responses, resistance to monotherapy of KRAS inhibitors is eventually developed. Significant progress has been made in understanding the mechanisms of drug resistance to KRAS-mutant inhibitors. Here we review the most recent advances in therapeutic approaches and resistance mechanisms targeting KRAS mutations and discuss opportunities for combination therapy.

## Introduction

Kirsten Rat Sarcoma Viral Oncogene Homolog (KRAS) mutations are genetic drivers in numerous cancer types including non-small cell lung cancer (NSCLC), colorectal cancer (CRC), and pancreatic ductal adenocarcinoma (PDAC) [1–5]. KRAS proteins primarily bind to guanosine diphosphate (GDP) and are in an inactive conformation maintained by intrinsic guanosine triphosphate (GTP) hydrolytic activity. KRAS interacts with GTPase activating protein (GAP) accelerating GTP toward conversion of GDP [6–9], while guanine nucleotide exchange factor (GEF) binding with KRAS results in the KRAS passively loading with the GTP [8, 10, 11]. GTP binding to KRAS shifts the active site from an open to a closed conformation, allowing multiple downstream effector pathways to interact and activate, including the mitogen-activated protein kinase (MAPK) and phosphatidylinositol 3-kinase (PI3K) pathways [12, 13]. The activated state of KRAS accumulating in vivo results in the activation of downstream signaling pathways and is associated with tumorigenesis, aggressive disease, and poor prognosis [14, 15].

For more than 40 years, KRAS mutation has been considered “undruggable” [16, 17]. On the one hand, the affinity of KRAS and GTP is at the pM level, while the concentration of GTP in cells is up to 0.5 μM. It is difficult to achieve effective competition like protein kinase inhibitors [18, 19]. On the other hand, the KRAS proteins are featureless. They have a nearly spherical structure that lacks a deep hydrophobic pocket and has no obvious binding site [20]. Currently, Food and Drug Administration (FDA) has approved an allele-specific covalent inhibitor of KRAS (G12C), AMG510 (sotorasib) having marked clinical responses across multiple tumor types [21–24]. In addition, a selective non-covalent inhibitor of KRAS (G12D), MRTX1133, also provides a novel targeting therapy [6, 22, 25]. In this new era of targeting KRAS mutations, the next challenge will be to understand and overcome the mechanisms of drug resistance.

In this review, we delineate the recent therapeutic strategies for KRAS mutant cancers and discuss the resistance mechanisms of KRAS mutant therapy and the possible approaches to combat them.

### KRAS mutation-driven cancers

**Fig. 1:**
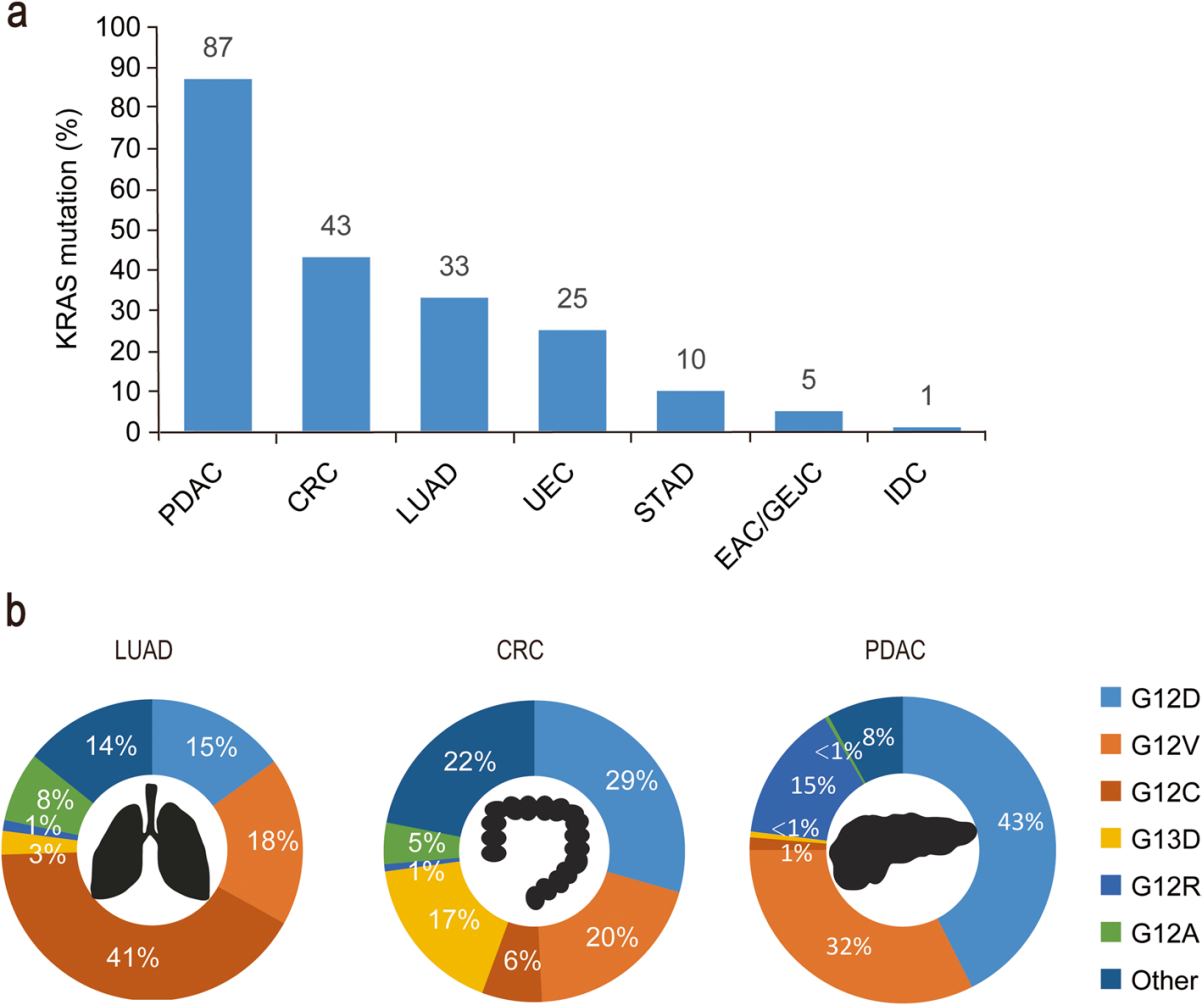
Types and proportion of KRAS mutations in multiple human cancers. **a** Mutations of KRAS occur in different types of cancers. Data derived from the AACR GENIE 9.0 public database. **b** Distribution of KRAS alleles in selected tumor types. The top 6 alleles with the highest overall disease rate are listed, while the other mutations were classified in the ‘other’ category. Mutation rates for KRAS were acquired from the Cancer Facts & Figs. 2000 report published by the American Cancer Society and published articles [26]. CRC, colorectal cancer; LUAD, lung adenocarcinoma; PDAC, pancreatic ductal adenocarcinoma; IDC, invasive ductal carcinoma; STAD, stomach adenocarcinoma; UEC, undifferentiated endometrial carcinoma; EAC/GEJC, esophageal adenocarcinoma/gastroesophageal junction cancer

KRAS mutations are common in a variety of cancers, for example, 45% of CRC cases in the United States and 49% of CRC cases in China; ∼90% of pancreatic ductal adenocarcinoma (PDAC) in the United States, and ∼89% in China; and 35% of lung adenocarcinomas (LUAD, a subtype of non-small-cell lung cancer) in the United States, and ∼13% in China (Fig. 1 for US data) [26]. KRAS has two isomers, KRAS4A and KRAS4B, that are generated by selective splicing of the KRAS gene. The mutant subtypes of KRAS are mainly classified as KRAS (G12D), KRAS (G12V), KRAS (G12C), KRAS (G13D), KRAS (G12R), and KRAS (G12A) mutations or KRAS wild-type amplification. Genetic alteration of G12 or G13 destroys the stability of the arginine residue hydrolysis transition state [7]. The distribution of KRAS mutations varies in different human cancers, with KRAS (G12C) mutation in 41% of LUAD, whereas KRAS (G12D) and KRAS (G12V) are the two most common alleles in CRC and PDAC, as shown in Fig. 1. Notably, other KRAS alleles such as G12R are limited in PDAC [26]. Indeed, although the tumor type is driven by KRAS mutations, its codons and the frequency of mutations vary by tissue type.

### KRAS biology: functions and signaling pathways

KRAS signaling provides a competitive advantage to cancer cells by participating in central carbon metabolism, increasing glucose uptake and glycolysis to increase the nutrients flux while promoting multiple branching biosynthetic pathways. It also regulates the total mitochondrial content and function by inducing phagocytosis, and the damaged mitochondrial delays tumor progression. KRAS also promotes alternative glutamine catabolism, leading to an increase in the production of nicotinamide adenine dinucleotide phosphate (NADPH) [27]. KRAS regulates pinocytosis to deal with the limited availability of mitochondrial substrates which can lead to dangerous levels of reactive oxygen species (ROS) and depleted nucleotide pools when cultured in buffered brine lacking essential nutrients. Autophagy flux provides KRAS-driven cancer cells with glutamine and glutamate to promote TCA cycling and support nucleotide production [28, 29].

Much more studies have revealed that KRAS is regarded as a switch for GDP-GTP regulation thus regulating the cytoplasmic signaling network and control various normal cellular processes. There are two splice variants of KRAS, KRAS4A and KRAS4B. In general, KRAS4B has higher expression levels. KRAS4A and KRAS4B are required for the initiation of tumor and may also have specific functions in tumor microenvironment. For example, KRAS4A expression increases tumor cell adaptation to stress, such as hypoxia. On the other hand, KRAS4B is expressed in both stem and progenitor cells. Recent studies have revisited the role of KRAS4A and KRAS4B in tumorigenesis [10, 30, 31].

As mentioned above, KRAS is a small guanosine triphosphatase (GTPase) that acts as a switch in the molecules of various cellular processes by coupling membrane growth factor receptors with intracellular signaling pathways and transcription factors. Combined with GTP, KRAS is activated, whereas KRAS is in the “off” state when binding to GDP [32]. KRAS activation is regulated by different negative and positive regulators. Negative regulators include GTPase-activating proteins (RAS-GAP), which enhances the inherent activity of KRAS-GTPase and leads to rapid hydrolysis of binding GTP [33]. Similarly, guanine nucleotide exchange factors (RAS-GEFs) are positive regulators that stimulate the release of bound GDP and exchange of GTP, resulting in the production of active KRAS-GTP complexes in response to upstream stimuli. The three main RAS-GEF families are SOS, RAS-GRF, and RAS-GRP. The SOS proteins are involved in downstream signaling transduction of receptor tyrosine kinases (RTKs), and the RAS-GRF protein is involved in Ca^2+^ influx/calmodulin-dependent activation of RAS and is mainly expressed in the central nervous system. The RAS-GRP activates RAS proteins downstream of non-receptor tyrosine kinases that are mainly expressed in hematopoietic cells [34].

Upstream signaling pathways of KRAS mainly include cell surface receptors, such as epidermal growth factor receptor (EGFR (ERBB1)), human epithelial growth factor receptor 2 (HER2 (ERBB2)), HER3 (ERBB3), and ERBB4. They transmit signals through KRAS after receiving external signals. This process stimulates cell proliferation and migration [35, 36].

Downstream signaling pathways mediated by KRAS mainly include rapidly accelerated fibrosarcoma (RAF) - mitogen-activated protein kinase kinase (MEK) -extracellular regulated protein kinases (ERK) and PI3K-serine/threonine-protein kinase (AKT) - mammalian target of rapamycin (mTOR) pathways. In the RAF-MEK-ERK pathway, activated KRAS-GTP rapidly increases the number of serine/threonine-specific protein kinase (RAF) from the cytoplasm to the plasma membrane, and then changes its conformation to activate it. The c-terminal catalytic domain of RAF binds to MEK1/2 and then activates ERK1/2 by phosphorylation to regulate cell proliferation, differentiation, and migration [37]. In the PI3K-AKT-mTOR pathway, KRAS-GTP binds to the p110s site of PI3K, which activates PI3K and promotes the conversion of phosphatidylinositol 4, 5-diphosphate (PIP2) to phosphatidylinositol 3,4,5-triphosphate (PIP3). PIP3 promotes AKT phosphorylation by phosphoinositide-dependent kinase 1 (PDK1) and activates mTOR, thus affecting cell proliferation, protein synthesis, survival, metabolism, transcription, and other life activities [38, 39]. In short, mutations in KRAS disrupt the guanine exchange cycle, resulting in KRAS “locking” in the active GTP-binding state, thereby activating downstream signaling pathways.

### Therapeutics for KRAS mutant cancers

#### Inhibitors directly targeting KRAS

The KRAS mutant proteins that drive cancer development are highly similar in sequence and structure based on the structural, mutational, and biochemical data of Harvey-RAS (HRAS). Direct inhibitors are most likely to bind to the catalytic domain of KRAS [40]. Research on direct inhibition of KRAS mutations date back to the discovery of RAS-activated mutations in human cancer cells in the 1980s when RAS-activated mutations were found in human cancer cells. However, some studies have found that KRAS mutants can be targeted by heterogeneous sites, to develop covalent inhibitors of KRAS mutants. The discovery of inhibitors that selectively target KRAS (G12C) while preserving the wild-type or other mutant KRAS is a breakthrough in the research field [20, 24].

**Fig. 2:**
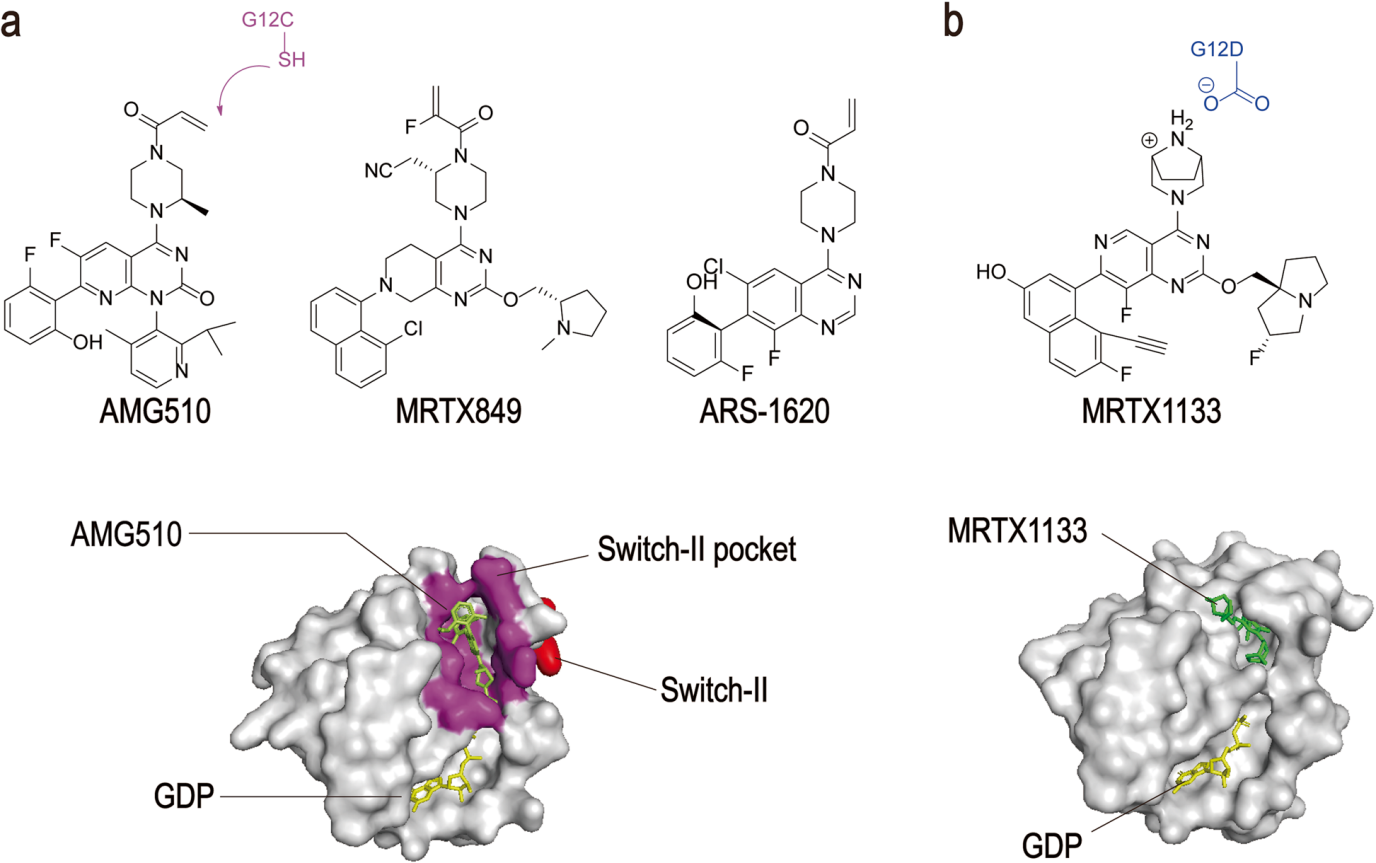
Structures of KRAS surfaces targeted by KRAS mutant inhibitors. **a** Switch-II pocket (purple) of KRAS (G12C) bound to AMG510 (PDB: 6OIM). **b** MRTX1133 with KRAS G12D/GDP (PDB: 7RPZ)

AMG510 is a first-in-class small molecule inhibitor of KRAS (G12C) that specifically and irreversibly locks KRAS in an inactive GDP binding state, as shown in Fig. 2 [41]. It was found that the potential inhibitor of KRAS (G12C) with pocket binding histidine-residues can be flipped upward to reveal hidden grooves produced by another orientation of His95. In contrast to the ARS-1620, the first confirmed direct inhibitor of KRAS (G12C), which occupies a smaller pouch and is less potent, AMG510 enhances its binding to KRAS (G12C) through the His95 groove with an approximately 10-fold increase in potency [42] (Table 1).

**Table 1:** Summary of KRAS mutation cancers therapeutics

Biomarker	Name	In vitro efficacy		In vivo efficacy			Ref.
		Tissue-Cell Line	IC_**50**_ (nM)	Dose (mg/kg)	Animal model	Inhibition% or Regression%^*a*^ (Day)	
**Act to directly inhibit KRAS**							
KRAS-G12C	ARS-1620	Lung-H358 (KRAS-G12C)	100	400	Pancreas-MIA-PaCa2 (KRAS-G12C)	-52 (18)	[21]
		Pancreas-MIA PaCa-2 (KRAS-G12C)	200	400	Lung-H358 (KRAS-G12C)	-33 (27)	[21]
	MRTX849	Lung-H2030 (KRAS-G12C)	0.2	100	Colorectal-CR6243 (KRAS-G12C)	-35 (20)	[43]
		Lung-H358 (KRAS-G12C)	2.5	100	Lung-Calu-1 (KRAS-G12C)	-81 (22)	[43]
		Pancreas-MIA PaCa-2 (KRAS-G12C)	29.6	100	Pancreas-MIA PaCa-2 (KRAS-G12C)	-100 (19)	[43]
		Lung-SW1573 (KRAS-G12C)	15.7	100	Lung-LU65 (KRAS-G12C)	-97 (13)	[43]
		Lung-H1792 (KRAS-G12C)	8.6	100	Lung-H1373 (KRAS-G12C)	-95 (22)	[43]
	AMG510	Pancreas-MIA PaCa-2 (KRAS-G12C)	1	100	Pancreas-MIA PaCa-2 (KRAS-G12C)	-56 (24)	[38]
		Lung-H1373 (KRAS-G12C)	5	100	CRC-PDX (KRAS-G12C)	-69 (41)	[38]
		Lung-H2122 (KRAS-G12C)	9	100	CRC-CT- 26 (KRAS-G12C)	-59 (29)	[38]
		Lung-H358 (KRAS-G12C)	3	100	Lung-H358 (KRAS-G12C)	-50 (24)	[38]
KRAS-G12D	MRTX1133	Stomach-AGS (KRAS-G12D)	6	30	Panc 04.03 (KRAS-G12D)	-70 (26)	[25]
**Targeted regulation of KRAS active protein**							
SOS1	BAY-293	Lung-H23 (KRAS-G12D)	734	–	–	–	[40]
		Colon-DLD1 (KRAS-G13D)	640	–	–	–	[40]
	BI-3406	Lung-H23 (KRAS-G12D)	9	50	Pancreas-MIA PaCa-2 (KRAS-G12C)	86 (22)	[41]
		Lung-H358 (KRAS-G12C)	24	50	CRC-LoVo (KRAS-G13D)	62 (23)	[41]
		Colon-DLD1 (KRAS-G13D)	24	50	CRC-B8032 (KRAS-G12C)	27 (22)	[41]
SHP2	SHP099	Lung-H23 (KRAS-G12D)	592	100	Oesophagus-KYSE520	98 (14)	[44]
		Lung-H358 (KRAS-G12C)	360	–	–	–	[44]
	TNO155	Lung-H3255 (EGFR-L858R)	120	7.5	Lung-H2030 (KRAS-G12C)	43 (14)	[45]
		Lung-HCC827 (EGFR-ex19del)	700	7.5	Esophageal cancer-KYSE410 (KRAS-G12C)	87 (14)	[45]
**Inhibitors of KRAS upstream signaling pathway**							
EGFR	AZD9291	Lung-H1975 (EGFR-L858R)	25	10	Lung-PC-9 (EGFR-ex19del)	-60 (14)	[46, 47]
		Lung-HCC827 (EGFR-ex19del)	> 250	10	Lung-H1975 (EGFR-L858R/T790M)	-68 (14)	[46, 47]
	JBJ-04-125-02	B cell-Ba/F3 (EGFR-L858R)	1000	100	Lung-H1975 (EGFR-L858R/T790M)	-70 (35)	[48]
**Inhibitors of KRAS downstream signaling pathway**							
MEK1/2	GSK112021	Pancreas-BXPC-3 (P53-mutant)	10	0.3	PDX-738	-20 (14)	[49]
ERK1/2	BVD-523	Pancreas-MIA PaCa-2 (KRAS-G12C)	500	100	CRC-Colo205 (BRAF-V600E)	-100 (14)	[50]
		Chromoma-A375 (BRAF-V600E)	500	100	Chromoma-A375 (BRAF-V600E)	-100 (18)	[50]
BRAF	BGB283	Chromoma-A375 (BRAF-V600E)	64	5	Colon-HT29 (BRAF-V600E)	75 (22)	[51]
		Colon-HT29 (BRAF-V600E)	50	10	Colon-Colo205 (BRAF-V600E)	> 100 (15)	[51]
p110α	BYL719	Medulloblastoma–DAOY (PIK3R1-mutant)	5650	25	Breast-MCF7 (PIK3CA-mutant)	-10 (21)	[52, 53]
AKT	MK2206	Liver-Huh7	3100	100	Pancreas-BT-474 (HER2-amplified)	70 (28)	[54]
mTOR	RAD001	Lymph-U937	20	–	–	–	[55]
	OSI-027	T cell-Jurkat	300	65	CRC-GEO	100 (12)	[56, 57]
**Degradation agent of KRAS**							
KRAS-G12C	LC-2	Lung-SW1573 (KRAS-G12C)	760	–	–	–	[58]
		Lung-H23 (KRAS-G12C)	250	–	–	–	[58]
**Small interfering RNA therapies**							
KRAS mRNA	AZD4785	Epidermal carcinoma-A431	10	50	Lung-NCI-H358	55 (28)	[59]

^*a*^1) Tumor growth inhibition was calculated when the mean final treated tumor volume was larger than the initial treated tumor volume using the following formula

Tumor growth inhibition = 100%*((Final vehicle tumor volume) – (Final treated tumor volume)) / ((Final vehicle tumor volume) – (Initial vehicle tumor volume))

2) Tumor regression was calculated when the mean tumor volume of the final treated tumor was smaller than the initial treated tumor volume using the following formula

Tumor regression = (−100%) * (1 – ((Final treated tumor volume) / (Initial treated tumor volume)))

MRTX849 (adagrasib) developed by Mirati Therapeutics, has been identified as a highly selective covalent inhibitor of KRAS (G12C) and is currently in phase I/II clinical studies [44, 45]. It is an oral, small-molecule selective inhibitor of KRAS (G12C) mutation. It can not only inhibit KRAS mutation almost completely in vivo but also show good drug-like properties. MRTX849 selectively targets the mutant cysteine 12 of KRAS in GDP, which is present in the induction-switch II pocket of KRAS (G12C), thereby locking it into an inactive GDP-binding state and inhibiting the RAS/MAP kinase pathway. In KRAS (G12C) positive cell lines and patient-derived xenograft models from multiple tumor types, 65% of the models showed significant tumor regression (Table 1) [60].

MRTX1133 was identified as a potent, selective, noncovalent inhibitor of KRAS (G12D) with picomolar binding affinity. Asp12 of KRAS (G12D) has a carboxyl group that is weaker nucleophilic than the sulfhydryl group of cysteine. This difference results in compounds, such as MRTX849, with significant effects on KRAS (G12C), but not on KRAS (G12D). Based on the structure of MRTX849, the electrophilic receptor, acrylamide, was replaced with piperazine to form intermolecular ion-pair force. MRTX1133 binds to the switch-II pocket and inhibits the protein−protein interactions necessary for the activation of the downstream pathway of KRAS [25] (Table 1).

#### Targeted regulation of KRAS active protein

The signal transduction process of KRAS activation and inactivation is catalyzed by various factors and enzymes. The KRAS activity can be indirectly reduced by inhibiting the function of factors or enzyme activity, which achieves the purpose of inhibiting pathway activation. Son of sevenless 1 (SOS1) and SH2-containing protein tyrosine phosphatase (SHP2) are the two most critical targets in the RAS signaling pathway [61, 62].

##### Molecules interrupting KRAS cell membrane localization or dimerization

It was observed that RAS is active when localized to the cell membrane. In this case, RAS requires three enzymes including isoprenylcysteine carboxyl methyltransferase (ICMT). Cysmethynil, a small molecule inhibitor of ICMT, disrupts RAS membrane binding and reduces cell growth in RAS mutant cell lines [46–48]. In addition, a small molecule, Cmpd2, which interferes with the binding of the RAS and lipid membranes, promotes membrane occlusion, and reduces binding to the RBD domain of RAF. What’s more, the RAS family members are oligomerized or dimerized for efficient RAS-driven signaling. Without interfering with RAS localization and GTPase activity, NS1 disrupts the self-binding of HRAS and KRAS by directly binding to the α4–α5 interface, reducing the activation of downstream pathways and inhibiting cell proliferation [30, 63, 64].

##### SOS1 inhibitors

In the RAS-GEF family, SOS protein is widely expressed and participates in downstream signaling transduction of RTKs. The human SOS family contains two different genes, SOS1 and SOS2, that are located on different chromosomes [65]. Hillig et al. found an effective and cell-active small molecule inhibitor, Bay-293, which is developed by Bayer and is currently in preclinical studies [61]. Bay-293 can effectively disrupt the interaction between KRAS and its exchange factor SOS1. It interrupts the reloading of KRAS and GTP by blocking the formation of the KRAS-SOS1 complex, leading to anti-proliferative activity [61]. The results showed that Bay-293 inhibited RAS activity in Hela cells and had high anti-proliferative activity against wild-type cell lines K562 and MOLM-13 and KRAS (G12C) mutant cell lines NCL-H358 and CALU-1 [61].

Hofmann et al. reported the discovery of a highly efficient, selective, and orally bioavailable small molecule SOS1 inhibitor BI-3406, which binds to the catalytic domain of SOS1 and thus prevents its interaction with KRAS [66]. BI-3406 reduces GTP-RAS formation, thereby limiting cell proliferation in a variety of KRAS-driven cancers. Most importantly, BI-3406 also attenuates MEK-in feedback reactivation, thereby enhancing the sensitivity of KRAS-mutant cancers to MEK inhibition [66].

##### SHP2 inhibitors

SHP2 is the first protein tyrosine phosphatase discovered to promote the development of cancer, which is closely associated with the occurrence of breast cancer and lung cancer. SHP2, as an oncogene, is located at the downstream common node of RTKs and mediates the activation of RAS-ERK signaling pathway, thereby promoting the proliferation of cancer cells. Currently, there are four SHP2 inhibitors in clinical trials: Jabi-3068, TNO155, RC-4630, and RLY 1971. These inhibitors bind to a region outside the PTP catalytic pocket and show great selectivity against other members of the phosphatase family [67].

Chen et al. reported an efficient, selective, and orally bioavailable small molecule SHP2 inhibitor, SHP099, which controls SHP2 in the self-inhibitory structure. SHP099 binds to the interface of N-terminal SH2, C-terminal SH2, and protein tyrosine phosphatase domains simultaneously, thereby inhibiting SHP2 activity through an allosteric mechanism. SHP099 inhibits the MAPK signaling pathway that results in inhibiting tyrosine kinase receptor-driven proliferation of human cancer cells in vitro and a mouse tumor transplant model in vivo [68].

The SHP2 inhibitor developed by Novartis, TNO155, inhibits MAPK signaling and enhances the efficacy of KRAS (G12C) inhibitors against KRAS (G12C) lung and colorectal cancers. The tumor microenvironment can be affected by blocking immunosuppressive signals RTKs and MAPK signals, thus reducing the overexpression of SHP2 and slowing down tumor growth [49].

#### Inhibitors of KRAS upstream signaling pathway

EGFR as an RTK plays a vital role in cell proliferation and migration. Most of the signaling transduction of EGFR is thought to occur in the plasma membrane, stimulating the activation and signaling transduction of MAPK and PI3K. Ligand-mediated EGFR activation is through conformational changes in the extracellular domain of the receptor following ligand binding, leading to receptor dimerization and the formation of kinase domains that internalize asymmetric dimers, while EGFR without ligand can also internalize, but at a relatively slow rate. Two classes of EGFR-targeting compounds, monoclonal antibody (mAb) like cetuximab, and tyrosine kinase inhibitor (TKIs) like gefitinib targeting the extracellular and intracellular domains of EGFR, have shown antitumor activities [69].

Gefitinib, reported by Mohamed Muhsin, is a first-generation small molecule EGFR inhibitor that binds to the intracellular tyrosine kinase domain of EGFR, thereby inhibiting autophosphorylation of receptor and then isolating the downstream signaling transmission [58]. Afatinib has clinical activity as a second-generation inhibitor against major uncommon and complex EGFR mutations in NSCLC [70].

AZD9291, reported by Cross et al., is a novel oral, potent, and selective third-generation irreversible inhibitor that inhibits EGFR (T790M) resistant mutation without affecting wild-type EGFR [71]. The Janne’s clinical trial of AZD9291 involved 253 patients. In 31 patients enrolled in the dose-escalation cohort, no dose-limiting toxic effects happened under the assessed dose [72]. An additional 222 patients were treated in five extended cohorts. The overall objective tumor response rate was 51%, indicating that AZD9291 was highly effective in patients with EGFR (T790M) mutant lung cancer whose disease had progressed during previous treatment with EGFR tyrosine kinase inhibitors [71, 72]. JBJ-04-125-02, as an EGFR-mutant allosteric inhibitor, inhibits EGFR (L858R/T790M/C797S) pathway and cell proliferation. However, increased dimeric EGFR reduces the efficacy, leading to drug resistance [73].

#### Inhibitors of KRAS downstream signaling pathways

##### RAF-MEK-ERK

Except downstream of post-translational KRAS and membrane-binding processes, other key targets are KRAS protein mutation-activated signaling pathways. One of these pathways is the RAF-MEK-ERK pathway, and several MEK inhibitors have been developed [74]. For example, trametinib (GSK112021) is a selective alloy structural oral inhibitor that inhibits MEK1/2 activation and kinase activity. Selumetinib, another oral MEK1/2 inhibitor, had no significant effect on survival compared to capecitabine in gemcitabine refractory PDAC patients. Lifafenib (BGB283) is a novel experimental inhibitor of RAF dimer with effective and reversible inhibition of the wild-type A-RAF, B-RAF, C-RAF, B-RAF^V600E^ as well as EGFR and KRAS. A dose-escalation/dose-expansion study in humans by Desai et al. evaluated the role of sorafenib in solid tumors with B-RAF and KRAS mutations. Antitumor activity was shown in KRAS-mutated NSCLC (response rate 16.7%) and endometrial/ovarian cancer (DCR 100%) [75].

Diamond et al. validated Cobimetinib, an oral inhibitor of MEK1 and MEK2 in a clinical trial. Among the 18 patients treated, the overall response rate was 89% and there was no acquired drug resistance. After 1 year, 94% of patients maintained the characteristics of progression-free tumors, demonstrating the efficacy of Cobimetinb in treating tumors by acting on the MAPK pathway [68].

Smetinib, another oral inhibitor of MEK1/2 developed by AstraZeneca, reduced the size of plexus neurofibroma (PNF) by at least 20% from baseline in 71% patients in the Phase 2 trial (SPRINT). In a Phase 2 trial with low-grade glioma (LGG), partial remission of PNF was found in 40% of patients and 2-year progression-free survival achieved in 96% of patients. In gemcitabine refractory PDAC patients, Selumetinib had no significant effect on survival compared with Cobimetinb [76, 77].

Ulititinib (BFD − 523) is an ERK1/2 kinase inhibitor with strong preclinical activity in cell lines with BRAF and RAS mutations. In phase I clinical trials, it has an acceptable safety profile and good pharmacokinetics, and is active against solid tumors with NRAS and BRAFV600 and non-V600 mutated cancers [78].

##### PI3K-AKT-mTOR

Another effector, PI3K, is also activated by KRAS. Unlike p110γ and p110δ, p110α is widely expressed and exclusively activated by RAS. As isoform-specific p110 inhibitors should target malignant cells more specifically, they are expected to have fewer off-target effects. Alpelisib (BYL719) is a p110α-specific inhibitor tested in a phase I trial including patients with PIK3CA-altered advanced solid tumors [10, 79].

MK2206, an AKT inhibitor, was found to improve the pathological complete remission (CR) rate in patients with breast cancer associated with positive hormone receptors [80]. Uprosertib (GSK795) is an ATP-competitive AKT inhibitor, which is evaluated in a phase I study involving patients with various advanced-stage solid tumors. The most frequent grade 3 or 4 drug-related adverse events were hyperglycemia (11%) and rash (3%), and partial remission (PR) was reported in one patient with anal cancer shown in preliminary safety and efficacy data [80].

Everolimus (RAD001), an allosteric mTOR inhibitor is another derivative of rapamycin approved by the FDA for the treatment of numerous cancers, such as advanced-stage renal cell carcinoma (RCC). In the phase III RECORD1 trial, everolimus was associated with prolonged progression-free survival (PFS) in patients with advanced-stage RCC [80]. OSI-027 targeting mTORC1/2 has been tested in a dose-expansion study involving 128 patients. The daily schedule uncovered 17% of patients experiencing grade 3 or 4 adverse events and six patients had stable disease for more than 6 months [81].

#### Degradation agents for KRAS mutant cancers

Xu et al. characterized a novel, selective AKT inhibitor MS21. MS21 degrades AKT to inhibit cell growth and maintain low signal transduction in PI3K-PTEN mutated cells. MS21 suppresses tumor growth in mice by depleting phosphorylated AKT (p-AKT) and the newly discovered AKT substrate AURKB in cells [82]. Bond et al. reported the first degrading agent LC-2 which is capable of degrading endogenous KRAS (G12C) [83]. It covalently binds to KRAS (G12C) and recruits E3 ligase VHL to rapidly induce KRAS (G12C) degradation and inhibits MAPK signaling in KRAS (G12C) mutated cancer cell lines. However, the safety evaluation system, pharmacodynamic studies, and dose selection of the proteolysis-targeting chimeras (PROTACs) technique need to be improved. The covalent nature of LC-2 may limit its potency because it fails to participate in catalytic rounds degradation, thus affecting the potency of LC-2 on cell viability [83] (Table 1).

#### KRAS mutant cancer vaccines

Vaccines may help body build an immune response to kill tumor cells and delay relapse. Vaccination allows the KRAS-mutant tumor antigen to cause T cell responses, which has become a promising treatment. Both the T cell pools of healthy individuals and cancer patients contain T cells that can identify KRAS mutation. After vaccination those T cells can be selectively expanded in tumor patients. Dendritic cells (DCs), one of the APCs, are specifically used to induce primary T-cell responses, and hence to induce antitumor immunity in vivo [84–86]. Purified peptide epitopes in combination with granulocyte-macrophage colony-stimulating factor (GM-CSF) induce efficient T-cell responses against peptide antigens in multiple cancers, including melanoma, breast cancer, and ovarian carcinomas [87, 88]. The cytokine GM-CSF promotes the maturation and activation of DCs, which can transfer to adjacent lymph nodes and activate effector T cells after antigen uptake [89]. In a phase I/II study, Gjertsen et al. have evaluated the immunogenicity and safety of KRAS peptide vaccine in 17 tumors with KRAS mutations. Two of the five patients with pancreatic cancer showed proliferative T cell responses, with a longer median survival of 10.5 months [90].

A second approach is an mRNA encoding a novel epitope of the KRAS mutation. Epitope discovery is an essential step in designing immunotherapies such as cancer vaccines. The conservative mutation profile of KRAS provides a valuable opportunity to develop neoantigen-targeted therapies. Clinical studies have shown effective CD4^+^ and CD8^+^ T cell responses to KRAS mutation tumors. Adham et al. validated the KRAS G12 mutation peptide as a bona fide epitope promoting the development of immunotherapy directed against this oncoprotein [91]. Chaft et al. revealed a clinical benefit in a patient with KRAS (G12D) metastatic CRC following the adoptive transfer of KRAS (G12D)-specific T cells restricted to HLA-C*08:02 [92]. With an intramuscular injection of an mRNA-containing vaccine, this mRNA nanoparticle is taken up by the antigen-presenting cells and translated to the cell surface, which results in the response of T cells [10].

#### Small interfering RNA therapies

Previous studies have shown that antisense oligonucleotides (ASO) are an attractive treatment for KRAS-driven human cancers, and therefore deserve further development. For example, Ross et al. demonstrated that delivery of nanoparticles containing small interfering RNA targeting KRAS mutations is an effective method. A chemically modified ASO, AZD4785, selectively and efficiently reduces the mRNA of KRAS in cells, thereby inhibiting the downstream effector pathways and exerting antiproliferative effects in KRAS mutant cells [59].

The LODERTM (Local Drug EluteR) developed by Silenseed Ltd., is a novel solution to the major challenges of addressing many diseases, including solid tumors. However, limitations include the delivery of RNAi-based drugs and prolonged activity at tolerated doses. siG12D-LODERTM aims to provide a local drug with prolonged activity within the tumor while ensuring that the siRNA drug is protected from degradation. It has shown its potential in phase I trial of combined chemotherapy in 12 patients with PDAC [93].

In this section, we describe recent advances in the development of therapies targeting KRAS mutation cancers, including directly targeting KRAS itself and RAS effector pathways, namely MAPK and PI3K. What’s more, we also expound emerging therapeutic strategies for treating KRAS-mutant tumors.

### Mechanisms of resistance to KRAS-mutant targeting therapy and combination therapy

#### Resistance mechanism of KRAS alterations and amplification

**Fig. 3:**
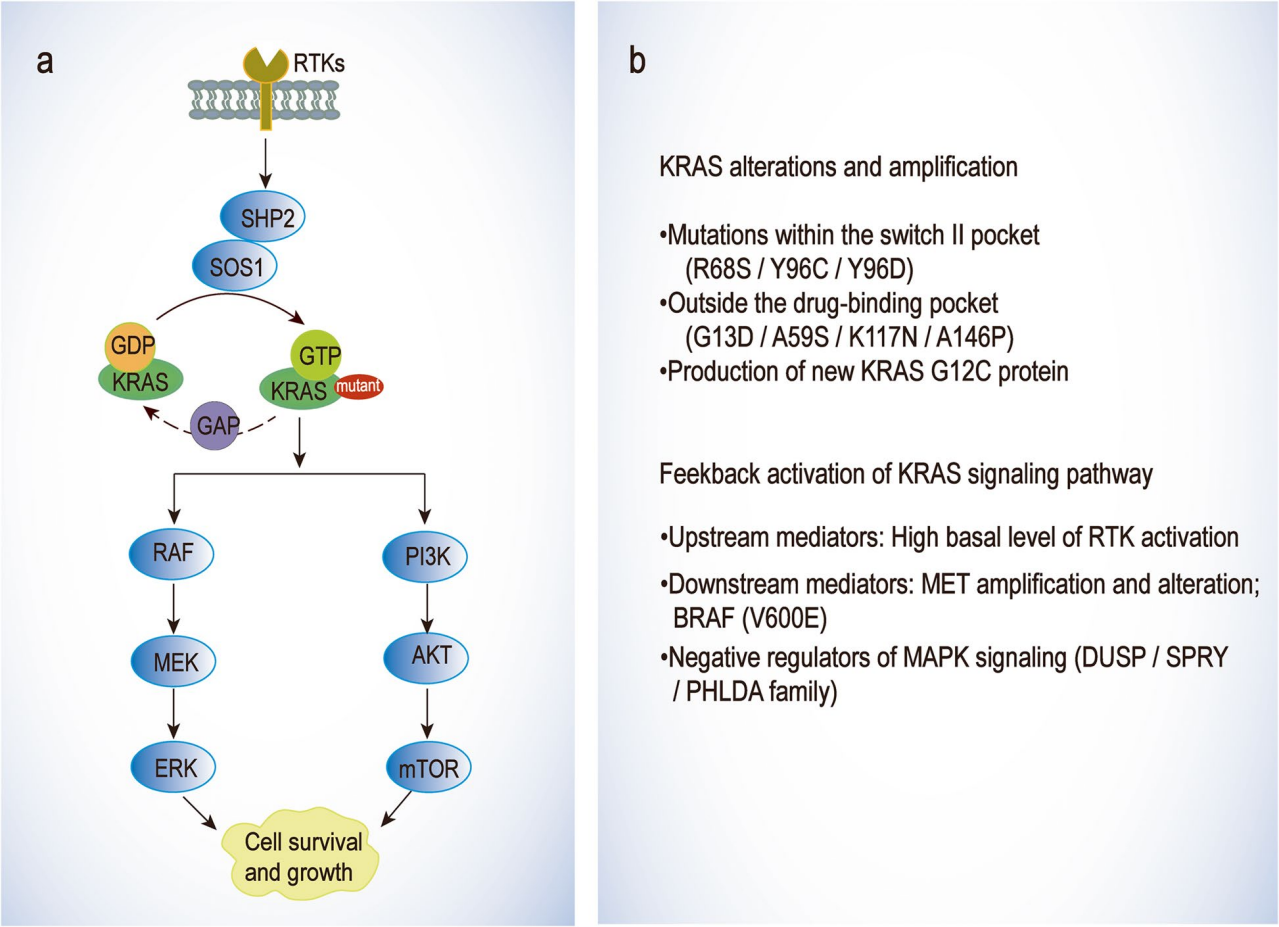
Overview of MAPK/PI3K signaling pathway and mechanisms of resistance to KRAS inhibitors. **a** Schematic representation of KRAS cycling and signaling pathway. **b** Mechanisms of resistance to KRAS inhibitors. Acquired mutations concerning the drug-binding sites and feedback activation of KRAS upstream and downstream signaling pathways favor drug resistance to KRAS mutant inhibitors

Mutations that disrupt covalent or potentially non-covalent drug binding can be used to illustrate clinical resistance to KRAS (G12C) inhibition. KRAS (R68S) and KRAS (Y96C) mutations are within the switch II pocket of the MRTX849 and AMG510 binding site. These mutations may disrupt drug non-covalent binding interactions. Awad et al. generated Ba/F3 cell lines with G12C/R68S, G12C/H95D, G12C/H95Q, G12C/H95R and G12C/Y96C double-mutant alleles and observed a marked resistance to MRTX849 in these cell lines [94, 95]. Recently, a novel KRAS (Y96D) mutation was found, which affects the switch-II pocket and reduces the H-bonding between the Y96 residue of KRAS and MRTX849. Thereby, KRAS (Y96D) conferred resistance to KRAS (G12C) inhibitors in patient-derived KRAS (G12C) xenografts [96]. RM-018, a neoteric KRAS (G12C) active state inhibitor, retains the ability to inhibit KRAS (G12C/Y96D) and may address the problem of drug resistance [96]. KRAS mutations, including G13D, A59S, K117N, and A146P, that are outside the drug-binding pocket and correlated with enhanced nucleotide exchange, are associated with milder drug resistance than G12R and Y96C mutations. These mutations may increase the portion of the active KRAS in a GTP-bound state that does not bind the drugs [94] (Fig. 3).

Awad et al. also observed some patients with high-level focal amplifications of the KRAS (G12C) allele [94]. Xue et al. found the increased new KRAS (G12C) protein in KRAS (G12C)-mutant tumor cells and elucidate that EGFR can promote the conversion of KRAS from the GDP state to the GTP-bound state (activated state) and AURKA can maintain its active state by binding to KRAS [7]. Since KRAS (G12C) inhibitors only bind to KRAS-GDP conformation, drug resistance may occur in cells with increasing GTP-bound KRAS [7]. Recently, novel molecules binding to the GTP-bound state of KRAS were discovered [11]. RM-007 and RM-008 covalently bind to KRAS (G12C) and KRAS (G13C), respectively, in the GTP-bound state and have antiproliferative activity in cells. However, since the canonical GTP confirmation could be disrupted after binding molecules, approaches targeting the GTP-bound state might also yield potential resistance to covalent inhibitors of GDP-bound KRAS (G12C) [10].

#### Feedback activation of KRAS upstream and downstream signaling pathways

Multiple studies have demonstrated that the efficacy of KRAS inhibition can be attenuated by feedback activation of upstream or downstream mediators and other negative regulators. Stimulation of ARS1620-treated cells with epidermal growth factor (EGF) resulted in reactivation of KRAS in the cells, strongly suggesting that EGFR mediates adaptive resistance to KRAS (G12C) inhibitors [97]. When activated by signals from RTKs, such as EGFR, KRAS triggered multiple proliferative signaling cascades, including MAPK and PI3K pathways to induce cell growth, division, and differentiation [8, 12, 98].

Further studies reveal that tumor type can affect response rates partially owing to the different levels of RTK activation. Most patients with KRAS (G12C) in NSCLC benefit from selective KRAS (G12C) inhibition, whereas CRC patients with the same mutation rarely respond to KRAS (G12C) inhibition [99]. Unlike NSCLC cell lines, the KRAS (G12C) CRC models have a high basal level of RTK activation and respond to growth factor stimulation [99]. KRAS (G12C) inhibition induces a higher rebound of phosphorylated ERK in CRC cells than in NSCLC cells. Hence, enhanced EGFR signaling is thought to mediate adaptive resistance of KRAS (G12C) inhibitors [99–101]. The anti-EGFR antibodies, cetuximab and panitumumab, were approved to treat RAS/RAF wild-type CRC. Cetuximab sensitizes the KRAS (G12C) CRC cells to AMG510, and the combination restores secondary resistance to anti-EGFR antibodies. In contrast, EGFR TKIs were approved for the treatment of EGFR-mutant lung cancer. Since RTK signaling in CRC is dominated by wild-type EGFR, a KRAS (G12C) inhibitor combined with an anti-EGFR monoclonal antibody can block receptor signaling rather than inhibit EGFR kinase activity [102–104].

Inhibiting KRAS mutation pathway by attenuating the activity of the upstream mediators is a promising combination therapeutic strategy. In addition to EGFR TKIs, BI1701963, a SOS1 blocker that acts as a pan-KRAS inhibitor, inhibits the binding of KRAS to GTP. BI1701963 is used in combination with trametinib to treat patients with any KRAS mutation [61, 97]. SOS1 inhibition in combination with AMG510 showed pharmacological blockade of WT RAS in KRAS (G12C) tumors. Furthermore, the combination of KRAS (G12C) inhibitor and WT RAS upstream activators, such as SHP2, is synergistic [105, 106]. SHP2 inhibitors can restore the sensitivity of KRAS-mutant NSCLC to MEK inhibition and increase inactive GDP-bound KRAS. A triplet combination of KRAS (G12C), MEK, and SHP2 inhibitors showed an augmented effect [7, 97, 107].

Meanwhile, the changing activity of the downstream effectors of KRAS also leads to the occurrence of drug resistance. For instance, BRAF (V600E) and MAP2K1/MEK1 (K57T, K57N, I99_K104 deletion, and E102_I103 deletion) can also cause acquired drug resistance [94, 108–111]. Sub-clonal evolution of MET amplification in KRAS (G12C) NSCLC cells that have become resistant to AMG510 in vitro has been reported previously [112]. Amplified MET increases the active form of RAS. Besides, MET also enhances AKT activation in the absence of RAS [112]. Criztinib is an MET inhibitor that restores sensitivity to AMG510 by eliminating the MAPK and PI3K signaling pathways. Dual MET/KRAS (G12C) inhibition resulted in tumor shrinkage in AMG510-resistant xenograft mice [94, 113]. Similarly, combining a KRAS (G12C) inhibitor with a PI3K or mTOR inhibitor could also overcome the adaptive increase in PI3K signaling in mouse xenografts [97, 114].

Besides the previously mentioned pathway molecules, negative regulators should be also taken into consideration. RNA sequencing revealed that KRAS (G12C) inhibition causes significant repression of DUSP, SPRY, and PHLDA family genes known as negative regulators of MAPK pathway [115, 116]. Oncogenic KRAS engages NF1/RSK1 to feedback inhibition of WT RAS signaling. Consequently, inhibition of oncogenic KRAS disengages this negative feedback pathway, leading to WT RAS activation and triggering adaptive drug resistance [105]. Taking together, inhibition of KRAS (G12C) can be overcome by feedback activation of either upstream or downstream molecules of KRAS. Therefore, the above findings of drug resistance mechanisms bring promising directions for combination therapy (Table 2).

**Table 2:** Clinical trials targeting KRAS mutation cancers

Target	Drug	Combinations	Tumor type	Phase	Trial number
KRAS-G12C	AMG510	Pembrolizumab (anti-PD-1 ab)	KRAS p.G12C Mutant Advanced Solid Tumors	II	NCT03600883
		MVASI	NSCLC	II	NCT05180422
		Docetaxel (microtubule inhibitor)	KRAS p. G12C Mutated Advanced Metastatic NSCLC	III	NCT04303780
	MRTX849	Docetaxel (microtubule inhibitor)	Advanced NSCLC	III	NCT04685135
		Pembrolizumab	Metastatic NSCLC	II	NCT04613596
		Cetuximab (anti-EGFR ab)	Malignant Neoplastic Disease	II	NCT03785249
	LY3499446	Abemaciclib (CDK4/6 inhibitor); Cetuximab (anti-EGFR ab); Erlotinib (EGFR inhibitor); Docetaxel (microtubule inhibitor)	NSCLC; CRC	II	NCT04165031
	JAB-21822	Cetuximab (anti-EGFR ab)	Advanced CRC	II	NCT05194995
	GDC-6036	Atezolizumab (anti-PD-L1 ab); Cetuximab (anti-EGFR ab); Bevacizumab (anti-VEGF ab); Erlotinib (EGFR inhibitor)	NSCLC; CRC; Advanced Solid Tumors	I	NCT04449874
KRAS-G12D	siG12D-LODER	Gemcitabine + nab-paclitaxel	Pancreatic Cancer	II	NCT01676259
SHP2	RMC-4630	LY3214996 (ERK inhibitor)	Pancreatic Cancer; CRC; NSCLC; KRAS Mutation-Related Tumors	I	NCT04916236
	ERAS-601	Cobimetinib (MEK inhibitor)	Advanced or Metastatic Solid Tumors	I	NCT04670679
SOS1	BI 1701963	Trametinib (MEK inhibitor)	Solid Tumors, KRAS Mutation	I	NCT04111458
ERK	GDC-0994	Cobimetinib (MEK inhibitor)	NSCLC; Metastatic CRC; Metastatic NSCLC; Melanoma	I	NCT02457793
	Ulixertinib	Pembrolizumab (anti-PD-1 ab)	Pancreatic Cancer	I	NCT03454035
p110α	GDC-0077	Entrectinib (pan-TRK inhibitor)	Advanced Unresectable or Metastatic Solid Malignancy	II	NCT04632992

*NSCLC* Non-small Cell Lung Cancer, *CRC* Colorectal Cancer

#### FAK-YAP Axis

**Fig. 4:**
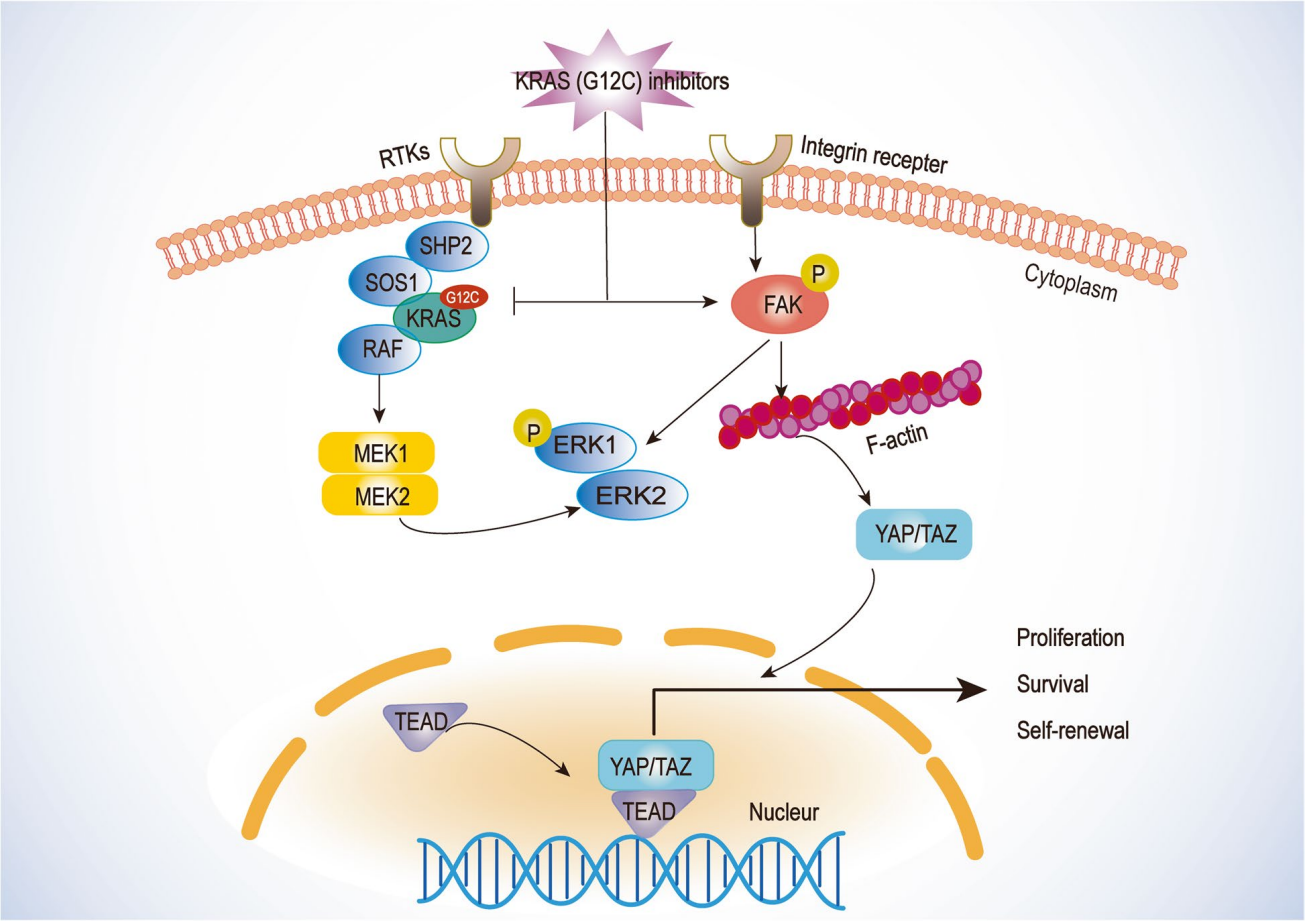
The schematic diagram of FAK-YAP axis affecting efficacy of KRAS (G12C) inhibitor

Focal adhesion kinase (FAK) is a non-receptor kinase that plays a role in regulating cell growth, signaling transduction, and tumor cell invasion [117]. Elevated phosphorylated FAK level is related to the poor prognosis of multiple cancers [118]. Multiple FAK inhibitors, such as IN10018, confer potent anticancer effects and effectively suppress the progression of KRAS mutant carcinoma [119–121]. FAK is a biomarker of the aberrant KRAS signaling pathway, and it responds to the administration of KRAS (G12C) inhibitors. Both aberrant FAK-YAP signaling and FAK-related fibrogenesis affect the development of resistance to KRAS (G12C) inhibitors. In short, sustained activation of FAK is induced by KRAS (G12C) inhibition, leading to attenuated treatment outcomes by dysregulating FAK-YAP signaling and fibrosis formation (Fig. 4). A synergistic effect was achieved with the combination treatment of KRAS (G12C) inhibition and a FAK inhibitor (IN10018). This combination simultaneously reduces the degree of drug resistance. The synergistic benefit of the combination therapy was consistently observed in different CDX and PDX models of KRAS (G12C) cancers [122]. Besides, activation of the FAK signaling pathway has an impact on the tumor microenvironment [123, 124]. FAK-related fibrosis can form a barrier in tumors that limits the CD8^+^ T cells infiltration in tumors, and FAK inhibition can ultimately promote the antitumor effects by decreasing the number of tumor-resident Tregs. Thus, FAK inhibition may enhance KRAS (G12C) inhibition and immunotherapy [125, 126].

#### Epithelial-to-mesenchymal transition (EMT)

Induction of epithelial-to-mesenchymal transition (EMT) is associated with intrinsic and acquired resistance to KRAS (G12C) inhibition in cell lines previously sensitive to AMG510. Induction of EMT leads to rewiring the expression of several RTKs, such as ERBB3 and FGFR1. Drug resistance induced by EMT enhances PI3K/AKT signaling and MAPK signaling [97, 127–129].

**Fig. 5:**
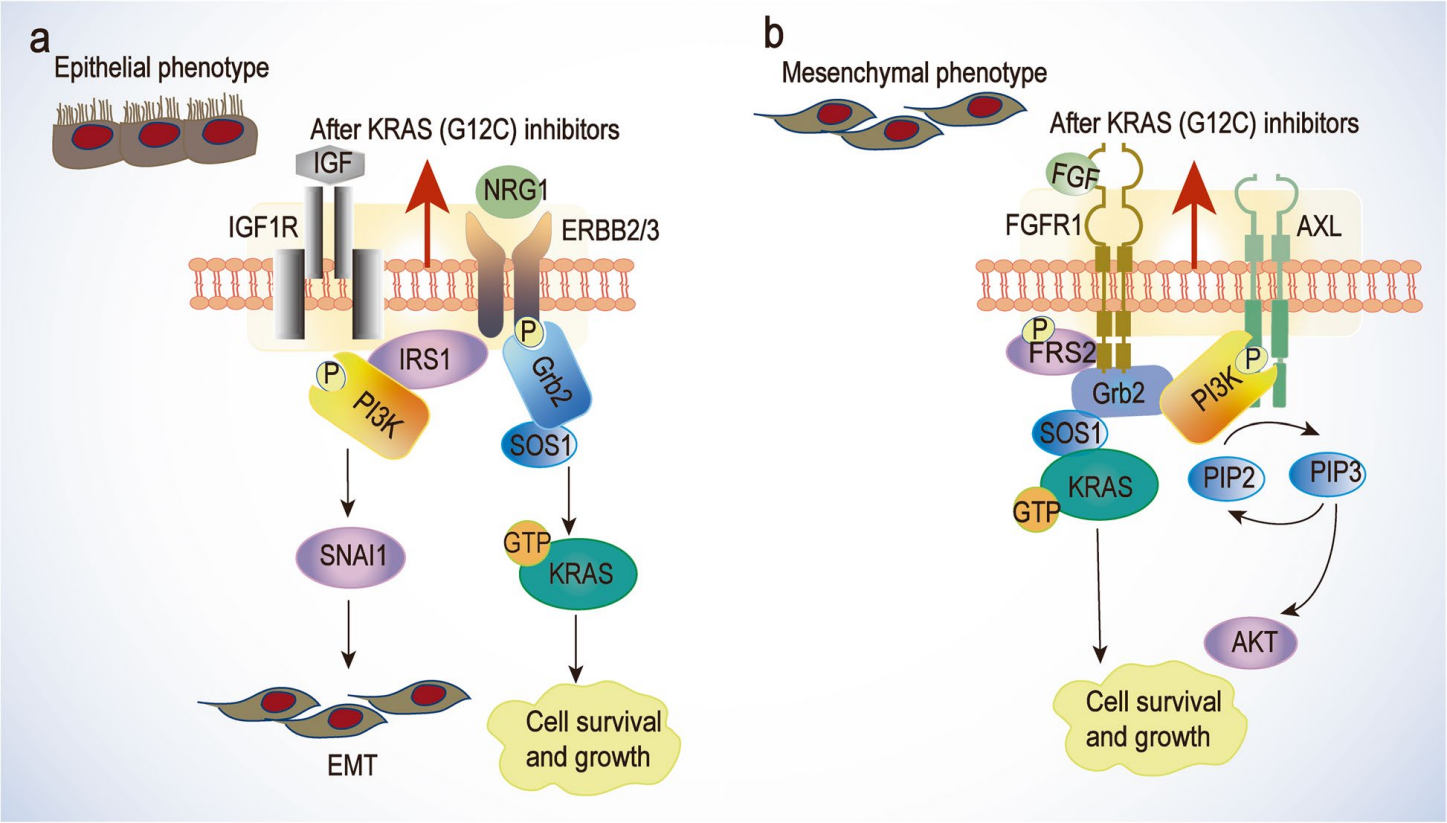
Adaptive responses to KRAS (G12C) inhibitors in epithelial and mesenchymal cells. **a** Inducing epithelial-to-mesenchymal transition (EMT) and cell growth in epithelial cell type. **b** FGFR1/AXL signaling in the mesenchymal cell type

Phosphoproteomics studies identified adaptive responses of cell types to KRAS-mutant inhibition [130, 131]. High basal ERBB2/3 associated with epithelial gene signatures was observed in KRAS (G12C) cell lines and human lung cancers. Markers related to IGF1R/ERBB2/3 pathway in the epithelial cells and fibroblast growth factor receptor (FGFR1)/AXL pathway in the mesenchymal cells should be considered in patient care. The IGF1R/ERBB2/3 signaling pathway may respond to the suppressed ERK and AKT signaling after KRAS (G12C) inhibitor treatment and favor the change to epithelial cell types (Fig. 5). This changed cell type is more sensitive to co-inhibition by SHP2 and SOS1 [130–135].

The above-mentioned drug resistance mechanisms can provide precise and effective treatment strategies through combination therapy. High feedback activation of the FGFR/AXL signaling pathway was found in mesenchymal cells. Inhibition of the FGFR signaling pathway reduces ERK and mTOR activation, whereas AXL inhibition attenuates the activation of PI3K pathway [43, 136–139].

#### The transition of pathological type

Awad et al. found that tumor cells of 2 out of 10 patients (9 with NSCLC and 1 with CRC) transformed from adenocarcinoma to squamous cell carcinoma during MRTX849 treatment without any other identifiable drug resistance mechanisms [94]. It uncovers that non-genotype resistance mechanisms exist. Similar phenomenon occurs in other targeted therapies for lung cancer [94, 140, 141]. Squamous cell differentiation has been described in the mechanism of acquired resistance to EGFR TKI treatment in lung adenocarcinoma. Adaptive changes in gene expression under treatment pressure may result in the conversion of one histological type to another one. The conversion of tumor cells into a distinct histological subtype leads to a loss of dependence on the original oncogenic driver as a mechanism of tumor escape from a targeted dependency. The therapeutic regimen for patients with lung squamous carcinoma transformation is not yet established and prospective studies are needed. Single-cell sequencing technologies will provide insight into this problem by confirming the histological subtype of tumor cell with molecular characteristics [142, 143].

#### Signaling of cell cycle regulation

Cyclin dependent kinase inhibitor 2A (CDKN2A), a cell cycle regulator and tumor suppressor, regulates CDK4/6-related RB phosphorylation and cell proliferation [60]. Loss of function mutation in the cell cycle tumor suppressor CDKN2A (p16) results in a hyperactivated cyclin-dependent kinases 4/6 (CDK4/6)-dependent retinoblastoma protein (RB) phosphorylation and a cell cycle transition. It was found that up to 20% of KRAS-mutant NSCLC has concurrent CDKN2A mutations. Hallin et al. illustrated that genetic alterations in cell cycle regulators resulted in cell cycle dysregulation and changed KRAS mutant allele frequency, identifying additional factors that could attenuate the therapeutic response to MRTX849 [60]. Because of the significant impact of MRTX849 on many genes in regulating cell cycle and apoptosis, a further understanding of the molecular mechanisms of its antitumor activity is necessary. Inhibition of cell cycle genes in vivo in MRTX849-treated xenografts intensified further tumor growth inhibition over the effects of KRAS inhibition alone [60]. Indeed, CDK4 inactivation led to reduced tumor development and induction of senescence in KRAS (G12V) mouse models. In a KRAS (G12C)-mutated and CDKN2A-deficient xenograft model, the combination of MRTX849 and the CDK4/6 inhibitor palbociclib resulted in a significant reduction in tumor volume, showing considerable synergistic effect [42, 144, 145].

#### Immune mechanisms

Histone deacetylase 5 (HDAC5)-induced bypass promotes macrophage recruitment into the TME and enables tumor recurrence following the extinction of KRAS (G12D) [146]. KRAS (G12D) signaling tightly controls transcription factors, including downstream effectors such as MAPK, PI3K/AKT, and the Janus kinase (JAK)/signal transducer and activator of transcription (STAT) signaling pathways that regulate HDAC5 expression. HDAC5 promotes the upregulation of Chemokine ligand 2 (CCL2) by inhibiting SOCS3, thereby recruiting CCR2^+^ macrophages [147]. These tumor-associated macrophages (TAMs) provide cancer cells with TGFβ that promotes KRAS mutation-independent PDAC cell growth after KRAS mutation-targeted inhibition. Inhibitors of the HDAC5-SMAD4 pathways in combination with KRAS (G12D) inhibition have synergistic antitumor effects in an isogenic PDAC model [146]. Using ingenuity pathway analysis to assess 950 differentially expressed genes, it was found that activated TGFβ signaling is a critical upstream mediator of different pathways related to drug resistance [148]. Consistent with changes in fuel source, there was a very significant increase in fat and bile acid metabolism as well as lipogenesis and myogenesis. In addition, tumors exhibited increased xenobiotics after treatment, suggesting that tumor cells are able to reduce intracellular AMG510 levels.

It has been shown that AMG510-resistant tumors have significantly reduced adaptive immune cell populations and become immunologically “cold” with dynamic and diverse remodeling patterns within the TME, such as alterations in angiogenesis, coagulation pathways, and fatty acid metabolism [149]. In almost all immunodeficient Balb/c mice, AMG510 induced only transient tumor regression followed by tumor relapse. It is revealed that the compromised host immune system may generate a novel drug resistance mechanism independent of MAPK reactivation [149]. The number of CD8^+^ T cells, macrophages, and DCs in KRAS (G12C) tumors increased significantly after 5 days of AMG510 treatment. Simultaneously, the expression of molecules for interferon signaling, chemokine production, and antigen processing increased [149]. Canon et al. suggested that AMG510 treatment resulted in increased T cell priming and antigen recognition and promoted the establishment of long-term T cell responses [42]. AMG510 treatment results in an inflammatory tumor microenvironment that is highly sensitive to immune checkpoint inhibition. Combining AMG510 with anti-PD-1 or anti-PD-L1 therapy augmented T cell infiltration and achieve longer-term efficacy [42, 50, 150].

#### Other resistance mechanisms in KRAS mutation cancers

Besides the resistance mechanisms mentioned above, there are many other resistance mechanisms deserving our discussion. For example, oncogene rearrangements, including EML4-ALK and CCDC6-RET, have been reported in three CRC patients treated with MRTX849 [94]. This observation could be explained by different genomic instability or DNA damage response at baseline or in response to KRAS inhibition in CRC and NSCLC. Fusion genes may also be one of the mechanisms of drug resistance [94]. Furthermore, Hou et al. revealed that USP21 increases macropinocytosis by microtubule-affinity-regulating kinase 3 (MARK3), thus providing metabolic support after KRAS mutation extinction in PDAC cancer cells [51]. USP21 overexpression also upregulates mTOR-related signaling pathways. Therefore, USP21 is expected to be a viable therapeutic target in PDAC, either as monotherapy or in combination with inhibitors targeting KRAS mutation [51, 52].

Another example of the resistance mechanisms is posttranslational modifications. KRAS is modified by SUMO3 in the conserved lysine42 reported by recent studies [53]. Dai et al. revealed that the expression of a SUMO-resistant mutant appears to suppress tumor growth in xenograft models [53]. Furthermore, sumoylation boosts tumor growth by maintaining KRAS (G12V) expression. The overexpression of wild-type KRAS significantly stimulated cell migration, which is further promoted by KRAS (G12V) [54, 55]. Combined, the observations of these resistance mechanisms help to develop new combination therapeutics overcoming clinical drug resistance [53].

In conclusion, the possible resistance mechanisms of KRAS (G12C) inhibition are diverse and complex. The main drivers include secondary mutations of KRAS itself, reactivation of multiple MAPK effectors from upstream and downstream of KRAS, immunodeficiency, etc. Combination therapy is currently an effective means to overcome drug resistance, and more cost-effective methods need to be explored.

### Future directions and conclusions

Specific KRAS (G12C) inhibitors will change the therapeutic landscape of KRAS-driven tumors, benefiting many patients with KRAS mutations [27, 56]. Unfortunately, innate and acquired resistance to KRAS inhibitors has hindered their development, rendering these new drugs less effective or even ineffective. In preclinical studies, possible resistance mechanisms for KRAS mutation therapy include secondary mutations in the KRAS binding site, reactivation of multiple upstream and downstream effectors, cell-cycle dysregulation, and immune deficiency [94, 95]. Importantly, these mechanisms of drug resistance appear to be tissue-specific [31].

It was thought that KRAS protein plays an on/off role in the GDP/GTP cycle. However, this concept oversimplifies the complex interactions between the states of individual molecules and the resulting dynamic protein conformations, which is unique to each mutation. The conformational state of each KRAS mutant determines the active state of the entire KRAS protein in the cells. Our current understanding of the impact of different mutations on the biochemical activity of KRAS proteins is based on the results of extensive structure-function studies conducted over the past 40 years, while biological validation of functional differences has only begun in recent years [57, 151].

Vetter et al. mention that different KRAS mutations are grouped into four categories based on their effects on GTP hydrolysis, nucleotide exchange, and effector protein interactions [152]. Tissue- and cell-type-specific factors such as extracellular signals, GAP and GEF expression and localization patterns, effector expression patterns and binding affinity, and cellular distribution of the two KRAS spliceosomes (KRAS4A and KRAS4B) should also be considered [31]. For example, KRAS (G13D) enhances nucleotide exchange. Rapid exchange mutants can synergize with GEF to produce extremely high levels of nucleotide exchange. However, these rapid exchange mutations may deplete the activity of GEF, thereby desensitizing cancer cells to GEF inhibition [152–154]. A more precise and accurate understanding of KRAS mutations is conducive to the development of novel inhibitors targeting KRAS mutations and the study of the corresponding drug resistance mechanisms.

Combination therapy based on a better understanding of drug resistance mechanisms holds great promise to induce long-term disease control or remission. Determining which combination strategies are most effective for patients will be challenging. First, tumor type can significantly affect response rates. According to the results of AMG510 in the phase I trial, this KRAS (G12C) inhibitor is effective in NSCLC (NCT03600883). 7 out of the 13 patients had a partial response (PR) and 6 had stable disease (SD). However, only 1 of 12 patients had a PR and 10 patients had SD in CRC [23]. Therefore, CRC is more difficult to treat than NSCLC, suggesting that CRC will require combination therapy. In phase I/II clinical trials (NCT03785249), three out of the five NSCLC patients achieved a PR, and one of two patients with CRC patients achieved a PR [94]. Moreover, combination therapy has always been more toxic, with poor safety, and combination therapy will bring more financial burden to patients [10]. The drug combination strategy should bring more efficacy and advantages to patients. Recently SHP2 inhibitors have shown promise in combination with KRAS (G12C) inhibitors. SHP2 inhibition increases GDP-bound KRAS levels, thereby enhancing the efficacy of G12C inhibition [107, 155]. In addition, the combination of AMG510 with anti-PD-1 therapy promotes T cell priming and long-term tumor-specific immune responses [42]. More studies need to be conducted to determine which combination strategy is most effective for patients.

## Data Availability

Not applicable.

## Abbreviations

AKT: Serine/threonine-protein kinase
ASO: Antisense oligonucleotides
CCL2: Chemokine ligand 2
CDK4/6: Cyclin-dependent kinases 4/6
CDKN2A: Cyclin dependent kinase inhibitor 2A
CRC: Colorectal cancer
DCs: Dendritic cells
EAC/GEJC: Esophageal adenocarcinoma/gastroesophageal junction cancer
EGFR: Epidermal growth factor receptor
EMT: Epithelial-to-mesenchymal transition
ERK: Extracellular regulated protein kinases
FAK: Focal adhesion kinase
FDA: Food and Drug Administration
GAP: GTPase activating protein
GDP: Guanosine diphosphate
GEF: Guanine nucleotide exchange factor
GM-CSF: Granulocyte-macrophage colony-stimulating factor
GTP: Guanosine triphosphate
GTPase: Guanosine triphosphatase
HDAC5: Histone deacetylase 5
HER2: Human epithelial growth factor receptor 2
HRAS: Harvey-RAS
ICMT: Isoprenylcysteine carboxyl methyltransferase
IDC: Invasive ductal carcinoma
JAK: Janus kinase
KRAS: Kirsten rat sarcoma viral oncogene homolog
LUAD: Lung adenocarcinomas
MAPK: Mitogen-activated protein kinase
MARK3: Microtubule-affinity-regulating kinase 3
MEK: Mitogen-activated protein kinase kinase
mTOR: Mammalian target of rapamycin
NADPH: Nicotinamide adenine dinucleotide phosphate
NSCLC: Non-small cell lung cancer
PDAC: Pancreatic ductal adenocarcinoma
PDK1: Phosphoinositol-dependent kinase 1
PFS: Progression-free survival
PI3K: Phosphatidylinositol 3-kinase
PIP2: Phosphatidylinositol 4,5-diphosphate
PIP3: Phosphatidylinositol 3,4,5-triphosphate
PR: Partial response
RAF: Rapidly accelerated fibrosarcoma
RB: Retinoblastoma protein
ROS: Reactive oxygen species
RTK: Receptor tyrosine kinase
SD: Stable disease
SHP2: SH2-containing protein tyrosine phosphatase
SOS1: Son of sevenless 1
STA: Stomach adenocarcinoma
STAT: Signal transducer and activator of transcription
TAM: Tumor-associated macrophage
UEC: Undifferentiated endometrial carcinoma
